# The effect of social grooming via live photo-sharing on well-being: the mediating role of social capital and moderating role of the need for privacy

**DOI:** 10.3389/fpsyg.2025.1627455

**Published:** 2025-07-11

**Authors:** Fei Wang, Xin Wang

**Affiliations:** ^1^School of Journalism and Communication, China West Normal University, Nanchong, China; ^2^School of Journalism and Communication, Chongqing University, Chongqing, China

**Keywords:** well-being, social grooming, live photos, social capital, need for privacy

## Abstract

**Background:**

Digital citizens in the social media era can share various types of photos. Live photo features on closed platforms, such as WeChat Moments, offer users a vivid sense of presence. However, the spontaneous nature of live photos may unintentionally reveal audio or visual details, raising privacy concerns. Previous research has not fully explored why users share live photos despite these risks, nor how live photo sharing affects users’ well-being from a social grooming perspective.

**Methods:**

We surveyed 266 users of WeChat Moments and analyzed the data using a moderated mediation model. Social capital was tested as a mediator between live photo-based social grooming and well-being, with the need for privacy as a moderator.

**Results:**

Social grooming through live photo sharing was positively associated with users’ well-being, and this association was mediated by social capital. Additionally, the strength of this positive association varied depending on users’ need for privacy. Specifically, a higher need for privacy was associated with a weaker relationship between social grooming and well-being, indicating that privacy concerns might constrain the potential benefits of social grooming.

**Conclusion:**

Social grooming via live photo sharing is associated with enhanced well-being through social capital, but the strength of this association depends on users’ need for privacy. To better balance social grooming and privacy protection, platforms and users should consider adopting flexible, user-controlled visibility settings.

## 1 Introduction

Social media has reshaped digital citizenship by reconceiving how individuals engage, interact, and form connections online. These digital interactions not only facilitate interpersonal communication but also redefine social relationships and impression management within digital communities. From Instagram’s Go Live and YouTube’s real-time broadcasts to TikTok LIVE and the live photo–sharing capacity in WeChat Moments, today’s social media landscape is dominated by dynamic visual formats. These formats cannot be edited after the shutter is pressed or the live-stream begins. However, while dynamic visual content delivers authenticity to audiences, it also raises the danger of inadvertent privacy leaks by accidentally capturing background conversations, workplace whiteboards, children’s faces, and precise location cues. The stakes are obvious on open, mass-audience services where a single misstep can reach millions (Duong et al., 2024; Trifiro, 2023). Similar risks also persist in seemingly safer, closed environments such as WhatsApp Status and WeChat Moments. Because closed platforms wrap non-editable dynamic visual content in a surface of exclusivity, users may feel even more exposed when accidental leaks occur among close contacts whose opinions matter most. Since editing after posting is impossible, impression management shifts from post-production editing to selective capture (choosing which moment to record) and audience control (deciding who may view the content and for how long) (Li et al., 2024; Ma et al., 2021; Zhang et al., 2022)—precisely the strategies that satisfy users’ need for privacy. To explain how people manage the balance between the social benefits of raw, unedited sharing and the privacy risks involved, the present study focuses on a typical closed network and examines how users’ need for privacy shapes the well-being gains they derive from live-photo–based social grooming.

Live photos are one of the non-editable visual content types shared on social media. A live photo is a hybrid of a still picture and a short video clip, which records a 1.5-s video snippet around the moment of capture, automatically bundled and presented as a single moving image (Kulkarni et al., 2024). Unlike ordinary photographs, live photos capture ambient motion and sound, offering a richer, more nuanced visual narrative and they cannot be edited or muted afterward. This blurs traditional boundaries between photography and videography, challenging the assumption that photographs are silent by embedding sound automatically (Zhao, 2025). User interactions differ in three ways: (a) live photos are easy to capture, requiring just one click, making the experience similar to simple photography rather than deliberate filming; (b) editing options afterward are minimal because the micro-clip cannot be rearranged or muted; and (c) viewers might be unaware of the embedded motion or audio, increasing the risk of accidental disclosure (e.g., private conversations, location cues). These features make live photos a new way to study visual self-disclosure in social grooming research.

A closed platform refers to a social network environment where content sharing is limited to mutually accepted contacts and detailed visibility controls (e.g., view lists, time-limited access). In China, WeChat (Tencent, Shenzhen, China) is one of the most popular closed platforms, with 1.402 billion monthly active users in 2025 (Tencent, 2025). WeChat Moments illustrates this architecture: posts on the platform are visible only to users mutually accepted as contacts, cannot be re-shared outside that circle, and can be retroactively hidden from subsets of one’s network (Li et al., 2024; Zhang et al., 2022). Such affordances shape grooming practices in ways that diverge from open platforms like Weibo and Instagram’s public feed.

On open networks, grooming often supports reach maximization and impression broadcasting; on closed networks, it shifts toward relational maintenance and trusted-circle intimacy. In other words, on open platforms such as Instagram’s public feed, senders accept weak-tie or stranger audiences, and manage privacy largely through selective omission. However, closed networks like WeChat Moments involve the known audience—every audience is a confirmed contact—so an unintended disclosure is judged by people whose opinions matter most. This may lead to privacy issues: the same leak that would be ignored by strangers can damage trust or cause embarrassment among strong ties, making privacy management even more central. In addition, the non-editable, motion-filled nature of live photos re-introduces uncertainty even within closed boundaries: accidental background revelations can disrupt established privacy expectations, and the relationship risks are arguably higher because the audience consists of close others whose evaluations carry greater emotional importance. Consequently, privacy management becomes not only relevant but central to understanding social grooming on closed platforms; users may balance the desire for authentic connection with the heightened risk of a privacy violation.

Digital social interactions through live photo–sharing on closed platforms introduce a social grooming model (Lin, 2019; Lin et al., 2024) in a digital environment. Where primates establish trust by removing one another’s fleas, today’s social media users nurture relational bonds by posting unscripted moments of their day and acknowledging others’ clips with quick taps of “like” buttons or by adding affiliative emojis in the comment thread (Lin and Hsieh, 2021; Liu and Yeo, 2024). Because live photos are captured and uploaded with a single action, they satisfy the need for the low-effort yet high-frequency contact that sustains intimacy in dense personal networks. In this sense, live photo grooming functions as an always-on maintenance ritual, signaling availability and emotional attunement without requiring lengthy text or carefully editing images. Although live photos limit later edits, users still manage impressions by deciding what to show and who may see it. As such, it may be an adaptive privacy management process.

A growing body of evidence links digital social interaction to variations in positive affect, life satisfaction, perceived social support and well-being (Choi and Noh, 2020; Hall, 2025; Li et al., 2021; Ostic et al., 2021; Zhao, 2023). For example, users’ social grooming as a digital interpersonal interaction positively correlates with their well-being and social outcomes. One study found that social grooming positively relates to users’ social capital, social connection, and social support (Lin and Hsieh, 2021; Liu and Yeo, 2024). Elsewhere, Lin (2019) reports that social grooming, including self-disclosure, relationship maintenance, and discussions of public topics, is positively associated with social capital and well-being. Further, Lin and Hsieh (2021) show how users’ social grooming has evolved over time, with strategic social grooming fostering enhanced social connections and improving well-being. Liu and Yeo (2024) demonstrate that social grooming increases life satisfaction among older adults by mediating between social capital and support. However, previous literature focusing on open social media aggregates types of self-disclosure, such as status updates, short videos, and static photographs, into a single concept of social grooming. Thus, it remains unclear how a new type of grooming (i.e., live photos as dynamic images) influences users’ social benefits. In other words, little is known about whether the same benefits arise, or are constrained, when the grooming object is a non-editable live image shared on a platform with mutually confirmed contacts such as WeChat Moments.

Any kind of posting on social media involves some degree of self-disclosure (Frener et al., 2024), which in turn raises privacy concerns. On open platforms, this is usually viewed as a cost–benefit calculation: users assess potential relationship benefits against privacy risks. Users with high privacy self-efficacy carefully select what to share (Lin et al., 2024). However, this cost–benefit view neglects the characteristics of closed platforms, where features like specific audience selection (e.g., sharing only with family, close friends, or colleagues) and time-limited viewing help reduce unintended privacy risks. Such platform features decrease concerns about privacy leakage during sharing and support continuous user engagement. Therefore, privacy protection and self-disclosure could exist together, rather than being mutually exclusive. Building on this insight, recent scholars consider privacy as a need rather than a mere transactional cost (Frener et al., 2024). Echoing Altman’s (1975) notion of boundary regulation and Petronio’s Communication Privacy Management theory (Petronio and Child, 2020), the need for privacy encompasses informational, psychological, and physical dimensions and emphasizes continuous boundary negotiation over one-off risk assessments (Dienlin, 2023). Applied to closed-platform live photo–sharing, this perspective explains why users dynamically adjust disclosure: by satisfying their need for privacy, they can enjoy social connection while minimizing risks (Schlosser, 2020), whereas excessive caution may heighten anxiety (Wang and Vergeer, 2024) and blunt well-being gains (Stevic et al., 2022). Nonetheless, audiences can still screen-record, breaking the supposedly sealed boundary—an affordance gap that places additional cognitive load on senders, who must weigh intimacy against potential risk. Accordingly, progress in gaining social benefits may be moderated by users’ degree of need for privacy. A higher level of the need for privacy in live photo–sharing may reduce the expected well-being gains.

To balance the social benefits of live photo social grooming with its privacy concerns, this study surveyed 266 WeChat Moments users. We tested a moderated-mediation model in which live photo grooming on WeChat Moments is positively associated with well-being. This relationship is mediated by social capital, and the magnitude of this benefit depends on users’ dispositional need for privacy. By focusing on a hybrid visual form within a genuinely closed network, we answer calls for finer-grained differentiation between content types, social capital, and privacy man agement processes, thereby integrating social benefits with social grooming within a closed-platform context. This study lays the groundwork for evidence-based design principles that can extend beyond the Chinese context to a platform where going live meets keeping it close.

This study offers three theoretical and two practical contributions. Theoretically, it (1) validates that live photo exchanges fit within the relational logic of the Social Grooming Model; (2) integrates communication privacy management by showing how a stable need for privacy moderates grooming’s link to well-being; and (3) situates these dynamics in a closed network context, thereby extending social media scholarship beyond the open-platform focus that dominates prior work. Practically, the results underscore the importance for users of adjusting audience scope and post-capture review to match personal privacy comfort and the need for designers to provide specific, post-posting visibility controls so that moment-to-moment sharing can strengthen close ties without damaging psychological boundaries.

## 2 Theory

This section introduces this study’s theoretical framework; specifically, Section 2.1 discusses the social grooming on well-being, Section 2.2 explores social capital, and Section 2.3 explores need for privacy.

### 2.1 Impact of social grooming on well-being

Social grooming in social media contexts refers to the purposeful, relationally oriented, adaptive self-disclosures and responsive interactions whose primary aim is to initiate, maintain, and strengthen interpersonal ties. Originating from primate research, where physical grooming strengthens affiliative bonds (Dunbar, 1991; Nakamura, 2003), the concept now encompasses digital behaviors such as posting daily updates; attaching live photos; and liking, commenting, or using emojis to signal attention and care (Donath, 2007; Lin, 2019; Lin et al., 2024; Lin and Hsieh, 2021; Liu and Yeo, 2024; Takano and Ichinose, 2018). Its key attributes are (1) intentionality, i.e., disclosures and reactions are performed with a clear social motive rather than merely broadcast without reason; (2) interactivity, where actions serve to invite or acknowledge feedback, creating an exchange loop that reinforces mutual awareness; and (3) tie-strength modulation, in that frequency, depth, and tone of grooming cues adjust in proportion to relationship closeness.

Users strategically engage in social grooming by carefully choosing what, when, and with whom they share content, aiming to balance social benefits with privacy risks. Social interactions on social media involve users actively communicating their thoughts, feelings, and experiences, rather than passively viewing content (Lee et al., 2023). Users intentionally express themselves and interact with others to build and maintain social connections and accumulate social capital (Chong and Choy, 2018; Ostic et al., 2021). These purposeful interactions—such as clicking “likes,” commenting, and messaging—are conceptualized as social grooming, as they visibly signal attention and care in digital environments (Tenenboim, 2022). Therefore, general social interactions and maintenance behaviors online can be seen as components of social grooming (Lee et al., 2023; Lin et al., 2024). Conceptually, social grooming encompasses strategic actions such as sharing personal, informational, emotional, public content, responding to others, and actively managing these interactions to enhance relationship quality (Lin and Hsieh, 2021; Menon, 2022). Operationally, it is measured through the frequency of posting, depth of self-disclosure, comment engagement, and the relational intentions behind these actions (Lin, 2019).

Empirical studies show that social grooming impacts users’ well-being (Lin et al., 2024; Lin and Hsieh, 2021; Liu and Yeo, 2024). Well-being refers to the evaluation of one’s positive affect, self-realization, and personal relationships with others from both hedonic and eudaimonic perspectives (Ryff and Singer, 2008), which means how people feel and how they function on both personal and social levels and how they evaluate their lives as a whole (Jarden and Roache, 2023; Michaelson et al., 2012). Positive social grooming on digital platforms enhances well-being (Raza et al., 2020; Zhao, 2023). Engagement in social grooming, understood as active, purposeful behaviors on social media to maintain and develop social ties, can lead to higher levels of life satisfaction (Lin, 2019; Takano and Ichinose, 2018). Community-building activities, ranging from friendly chats to the exchange of personal narratives, foster a stronger sense of belonging and support, leading to greater well-being (Guo et al., 2014; Zhan et al., 2016). Grounded in the structural–functional model of social support (Liu and Yeo, 2024), such grooming interactions serve as a form of structural integration, enabling users to tap into supportive exchanges that ease everyday stressors. Individuals who self-disclose in a supportive environment and exchange small emotional gestures or encouraging remarks tend to report a greater sense of well-being in their lives (Burke and Kraut, 2016).

Most previous studies have focused on text-based and static image posting, not dynamic image posting. In static images, users can carefully select and edit what they want to disclose (Kim and Chock, 2015; Lin, 2019). Today, new forms of live photo content offer a less-scripted, more realistic view of personal moments. Therefore, social grooming via live photo–sharing, compared to static image posts and well-edited videos, may also generate a sense of authenticity by capturing unplanned details of real life, helping to maintain and strengthen social relationships (Lin et al., 2024). As a result, social grooming via live photo–sharing may strengthen social ties, promote social connections, and improve users’ well-being. Based on the above reasoning, we expected that hypothesis 1 (*H1*): *Greater social grooming via live photo–sharing is positively related to individuals’ well-being.*

### 2.2 Social capital

Social capital refers to the resources made available through an individual’s social relationships and networks, accumulated over repeated interactions (Guo and Chen, 2022). Social capital consists of resources embedded in one’s social networks—resources that can be accessed or mobilized in purposeful actions through ties in the networks (Lin et al., 2001, p. 29). Social media usage (Fenton et al., 2023; Ghorbanzadeh et al., 2023; Guo and Chen, 2022; Kim and Kim, 2021; Ostic et al., 2021; Soh et al., 2022) and social grooming behaviors (Lin et al., 2024; Liu and Yeo, 2024) may positively relate to social capital. People gain higher social capital by managing social impressions on social media (Tuominen et al., 2022). One of the ways to build social impressions is by sharing photos (van der Zanden et al., 2022). When people want to make a good impression on their audience (Sezer, 2022), social grooming via live photo–sharing may be useful because providing live photos can strengthen users’ social connections with close friends, reinforce users’ group bonds, expand networks of users’ acquaintances, and unlock users’ supportive resources (Abel et al., 2021; Chen and Lemmer, 2024; Guo and Chen, 2022; Murari et al., 2024). Sharing these photos may yield benefit through stronger ties that provide emotional support or weaker ties that introduce different information and opportunities (Ahmad et al., 2023; Huang et al., 2021; Iantosca et al., 2024; Zhang and Sung, 2023). Thus, as social capital grows, individuals typically report improved mental health. As such, social connection through live photo–sharing may influence social capital, thereby improving well-being. Based on this reasoning, we expected that hypothesis 2 (*H2*): *Social capital mediates the positive relationship between social grooming and well-being.*

### 2.3 Need for privacy

The need for privacy refers to an individual’s cross-situational and temporally stable preference for access to the self or withdrawal from others (Frener et al., 2024). It is a higher-order psychological need, cross-situational motive to regulate access to the self—a stable preference that shapes, rather than merely reacts to, each disclosure event. Its theoretical roots lie in Altman’s (1975) Privacy Regulation Theory, which frames privacy as the selective control of informational, emotional, and psychical boundaries (Weber et al., 2021). Communication Privacy Management theory extends this insight by showing that boundary control is never purely individual but always negotiated with co-owners of information, making privacy management an ongoing relational process (Petronio and Child, 2020). In digital settings, this motive appears in behaviors such as hiding unintended backgrounds in live photos, restricting who may view a “story,” and steering conversations away from sensitive topics, all actions as communicative tools for achieving a exposure. Because the need for privacy is foundational to self-realization (Margulis, 2003), satisfying it enables more authentic disclosure and deeper intimacy (Trepte, 2021, 2023), whereas not meeting this need triggers withdrawal or content filtering (Meier et al., 2020). Unlike situational privacy concerns, which rise and fall with perceived risk (Dienlin, 2023), the general need for privacy persists across contexts, motivating users to employ active boundary strategies rather than abandon social media altogether.

Within the social grooming model, live photo–posting is valuable because it translates small disclosures into social capital and affective rewards (Lin, 2019). Yet, every disclosure simultaneously activates the boundary-negotiation processes described by Communication Privacy Management theory, which treats relational communication as a continual balancing of openness and protection (Petronio and Child, 2020). These two frameworks intersect most clearly in the closed-platform setting of this study: the richer and more spontaneous the grooming cue (e.g., an unedited live photo), the greater its bonding potential and the greater the need for rule coordination about who may see, save, or forward it. To integrate the models empirically, we positioned social grooming frequency as the pathway to well-being, communication privacy management’s boundary rules as the behavioral mechanism that can limit that pathway, and the need for privacy as the individual-level variable that sets the default strictness of those rules. In other words, grooming produces value, communication privacy management explains the transactional cost, and the need for privacy adjusts the cost–benefit ratio for each user.

The need for privacy may be a moderator of social grooming because it embodies its theoretical function as a stable motivational reference point (Frener et al., 2024). Grounded in Altman’s (1975) balance of desired versus achieved privacy and control-centered definition, this need dictates how restrictive communication privacy management rules must be before a person feels comfortable enough to groom. Users with a low need tolerate wider audiences and minimal editing, enabling grooming to flow freely into social capital; users with a high need invoke tighter filters, post-capture review, and content selection, which can weaken both the spontaneity and relational payoffs of grooming. This personal set-point systematically shapes when and how boundary rules are enacted, independent of situational risk evaluations (Dienlin, 2023). Therefore, need for privacy may serve as a boundary condition for explaining why grooming behavior yields different well-being outcomes across individuals. Based on the above reasoning, we proposed the following hypothesis 3 (*H3*): *The need for privacy negatively moderates the relationships between social grooming and (a) social capital and (b) well-being.*

**Figure 1:**
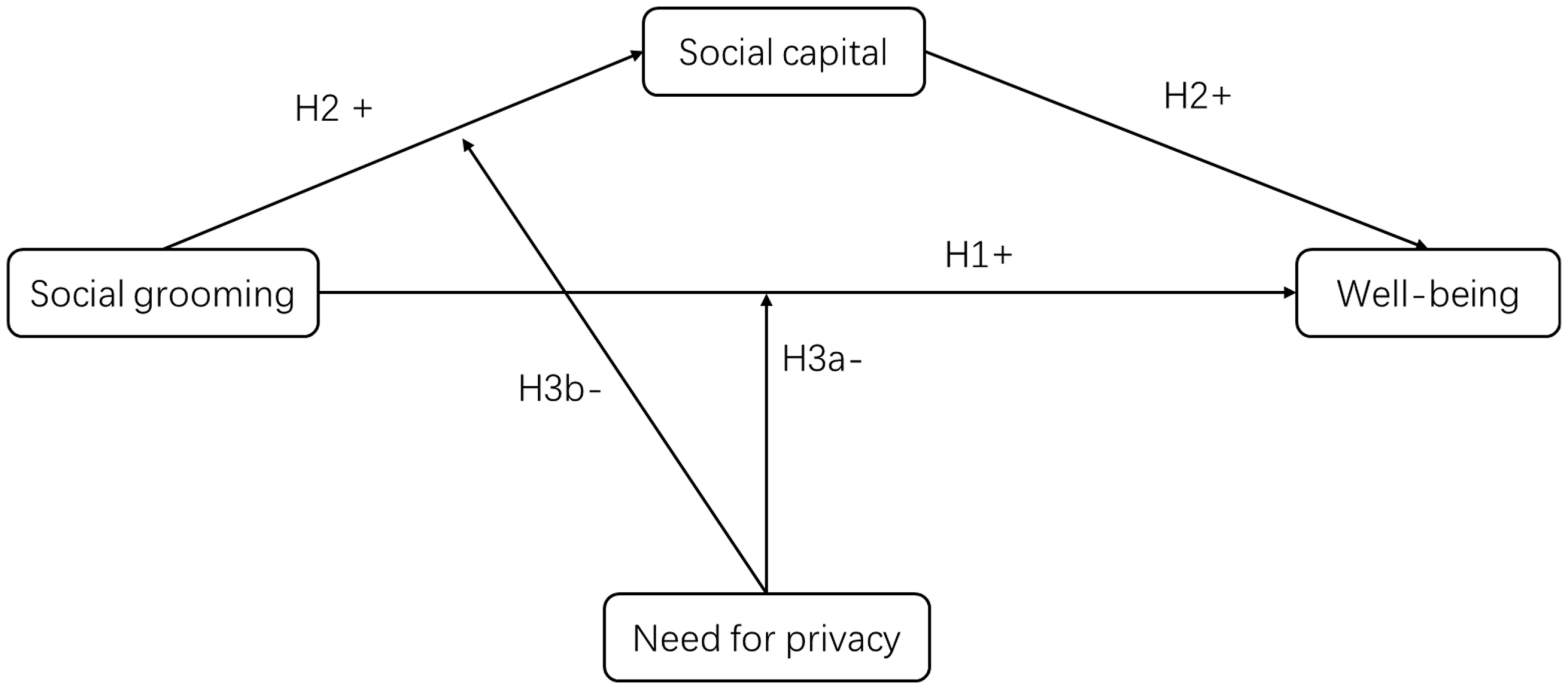
Theoretical model.

Figure 1 shows the theoretical model of this study.

## 3 Methods

### 3.1 Participants and procedure

We selected WeChat Moments, a closed social media platform popular in China with a substantial user base, which launched live photo–sharing in September 2024. Ethical approval for this study was obtained from the ethical review committee of the School of Journalism and Communication, Chongqing University (approval no. CQUXWXY202412002).

Participants were recruited using the Wenjuanxing online survey platform (Chinese company, https://www.wjx.cn/). Eligible individuals included Mandarin Chinese-speaking active WeChat users aged ≥18 years who confirmed they had posted, viewed, and responded to at least one live photo entry in WeChat Moments during the previous month. Eligibility was confirmed through a screening questionnaire. A final valid sample (*N* = 266) was obtained. Participants provided informed consent, were assured of confidentiality, and were informed that data collected would be used exclusively for academic research purposes.

Among respondents, 40.23% (*n* = 107) were male and 59.77% (*n* = 159) were female, with an age distribution as follows: 3.76% aged 18–20 years, 21.80% aged 21–25 years, 30.83% aged 26–30 years, 39.47% aged 31–40 years, and 4.14% aged 41–50 years. Regarding educational background, participants held high school qualifications (1.13%), junior college qualifications (4.89%), bachelor’s degrees (87.97%), or postgraduate degrees or higher (6.02%). The sample was geographically diverse across China’s city-tier system, with 23.0% from tier 3 cities, 20.8% from new tier 1 cities, 17.7% from tier 2 cities, 14.3% from tier 1 cities, 12.8% from tier 4 cities, 9.4% from tier 5 cities, and 1.9% unclassified.

Since participants were Mandarin Chinese-speaking individuals, the survey was administered in Mandarin Chinese. To ensure measurement accuracy and equivalence, scales originally developed in English underwent rigorous forward and back-translation procedures. Two bilingual researchers independently translated and back-translated the scales to confirm equivalence and accuracy.

### 3.2 Measurements

All variables were measured using established scales adapted to reflect live photo–sharing activities on WeChat Moments. Each item was reworded to mention live photos explicitly and to anchor responses to WeChat Moments (see Supplementary material). Forward–back translation confirmed linguistic equivalence. Mean scores of the items formed composite indices.

#### 3.2.1 Social grooming

Social grooming (Cronbach’s *α* = 0.79, M = 3.02, SD = 0.64, KMO = 0.81, explained variance = 54.36%) was measured using a 5-item scale adapted from the work of Lin (2019). Participants rated how often they engaged in specific live photo–sharing behaviors on a 5-point scale (1 = never, 5 = very often), answering questions like *How often do you share personal emotions through live photo posts* (e.g.*, happiness, sadness, excitement*) *on your WeChat Moments?* (M = 3.12, SD = 0.83; Manago et al., 2012); *How often do you share daily general events with live photos (such as what you are doing, where you are, or what you are eating or listening to)*? (M = 3.24, SD = 0.77; Manago et al., 2012); *How often do you express opinions on controversial issues (e.g., nuclear power) through live photos?* (M = 2.44, SD = 0.99; Sleeper et al., 2013); *How often do you use live photos to discuss noncontroversial and trending topics (e.g., viral social media challenges, interesting news)?* (M = 2.98, SD = 0.92; Sleeper et al., 2013); and *How often do you respond to others’ live photo posts (e.g., liking, commenting, emojis)?* (M = 3.31, SD = 0.79; Ellison et al., 2014).

#### 3.2.2 Social capital

Social capital (Cronbach’s α = 0.80, M = 3.90, SD = 0.56, KMO = 0.84, explained variance = 50.01%) was measured using six items adapted from the work of Williams (2006), focusing on interpersonal support and connection. An example item is: *There are several people I trust to help solve my problems*. Participants responded on a 5-point scale (1 = strongly disagree, 5 = strongly agree).

#### 3.2.3 Need for privacy

The need for privacy (Cronbach’s α = 0.86, M = 3.07, SD = 0.66, KMO = 0.86, explained variance = 71.84%) was measured using four items per dimension, adapted from Frener et al. (2024). Items were rated on a 5-point scale (1 = strongly disagree, 5 = strongly agree); item examples include *I do not want my live photos or personal data publicly accessible*, *I feel uneasy when others reveal very private details through live photos*, and *I feel uncomfortable when people enter my room or check my photo album (including live photos) unannounced.* Higher average scores indicated stronger privacy concerns.

#### 3.2.4 Well-being

Well-being (Cronbach’s α = 0.85, M = 3.44, SD = 0.78, KMO = 0.79, explained variance = 68.40%) was measured using four items adapted from the work of Lin (2019). Participants responded on a 5-point scale (1 = strongly disagree, 5 = strongly agree) to items such as *You feel that you are closer to your friends through interactions of sharing live photos in WeChat Moments* (M = 3.36, SD = 0.91; Köbler et al., 2010); *Generally speaking, are you satisfied with your life?* (M = 3.47, SD = 0.96; Diener et al., 1985); *Generally speaking, are you satisfied with your social life?* (M = 3.42, SD = 0.95; Lin et al., 2012); and *Generally speaking, are you happy with your current life?* (M = 3.50, SD = 0.95; Keyes, 2009). Higher average scores reflected greater subjective well-being.

### 3.3 Analysis

**Figure 2:**
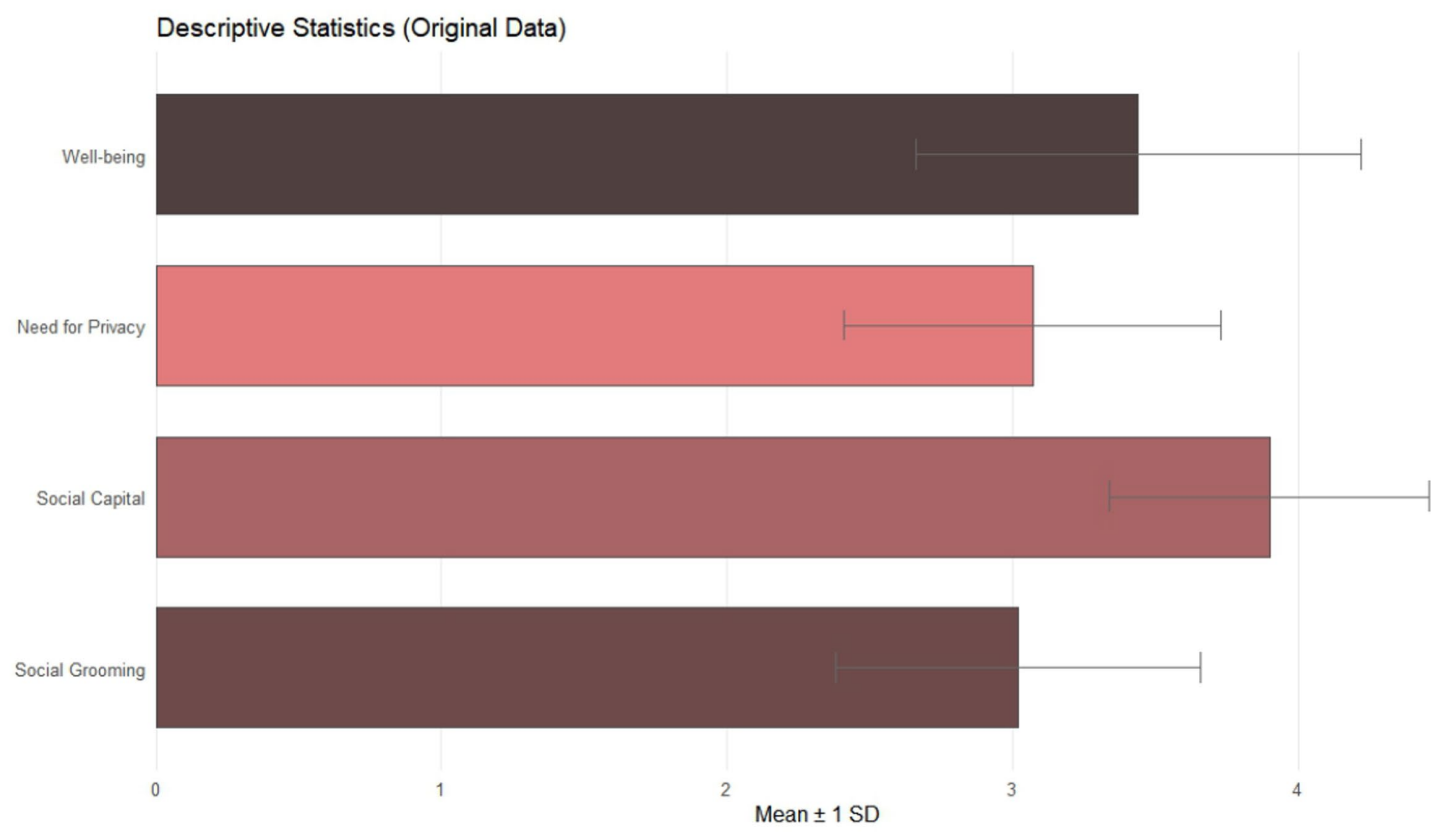
Descriptive statistics.

We employed SPSS (version 29; IBM Corporation, Armonk, NY, USA) to analyze data. We standardized original data (Table 1 and Figure 2 provide descriptive statistics). The distributional properties of each variable were deemed acceptable for standard parametric testing given that the absolute kurtosis values were all less than 10 and the absolute skewness values were all below 3. We used PROCESS Model 8 (Hayes, 2022) because our theoretical model proposes that the relationship between social grooming and well-being is mediated by social capital and moderated by the need for privacy. Bootstrapping was performed with 5,000 resamples to generate bias-corrected 95% confidence intervals for all estimates. The overall model yielded acceptable fit indicators, confirming its stability. No additional covariates were included in the final model.

**Table 1:** Descriptive statistics of all variables.

Variable	Cronbach’s alpha	Original scale				Zero-centered scale			
		Max	Min	Mean	SD	Max	Min	Mean	SD
Social grooming	0.79	5.00	1.00	3.02	0.64	1.98	−2.02	0	0.64
Social capital	0.80	4.83	2.00	3.90	0.56	0.93	−1.90	0	0.56
Need for privacy	0.86	4.50	1.25	3.07	0.66	1.43	−1.82	0	0.66
Well-being	0.85	5.00	1.50	3.44	0.78	1.56	−1.94	0	0.78

Cronbach’s alpha values indicate internal reliability of scales. “Zero-centered scale” refers to standardized variables (Mean = 0).

No control variables (e.g., age, gender, education) were included, as our theoretical framework does not propose that these demographic factors influence the hypothesized relationships. Model-fit indicators confirmed robust and stable results without these variables.

## 4 Results

### 4.1 Direct effect

H1 predicted that greater social grooming via live photo–sharing would positively relate to individuals’ well-being. As detailed in Table 2, the regression analysis yielded significant support for this prediction, showing a meaningful positive association (total effect: b = 0.40, *p* < 0.001, ΔR^2^ = 0.14). Hence, the data align with H1.

**Table 2:** Path analysis results.

Pathway	Effect	SE	t	p	95% CI Lower	95% CI Upper
Direct effects						
Social Grooming → Social Capital	0.32	0.05	6.74	<0.001	0.23	0.41
Social Capital → Well-being	0.29	0.09	3.22	<0.001	0.11	0.47
Social Grooming → Well-being (direct)	0.30	0.08	3.78	<0.001	0.14	0.46
Indirect effect (Mediation)						
Social grooming → Social capital → Well-being	0.09	0.03	–	–	0.03	0.16
Total Effect (direct + indirect)	0.40	0.08	5.18	<0.001	0.25	0.55

Confidence intervals (95% CI) indicate statistical significance when excluding zero.

### 4.2 Mediation effect

H2 proposed social capital as a mediator between social grooming and well-being. Results summarized in Table 2 indicate a significant positive relationship from social grooming to social capital (b = 0.32, *p* < 0.001, ΔR^2^ = 0.13) and from social capital to well-being (b = 0.29, *p* < 0.001). Furthermore, the bootstrap analysis confirmed a statistically significant indirect pathway (indirect effect = 0.09, 95% BC CI [0.03, 0.16]), providing empirical support for H2.

### 4.3 Moderating effects

H3 examined whether the need for privacy moderates the mediation process, hypothesizing that a higher need for privacy would attenuate the associations of social grooming with social capital (H3a) and well-being (H3b). The interaction term between social grooming and need for privacy was not statistically significant when predicting social capital (b = −0.10, *p* = 0.15), thus failing to support H3a. However, as depicted in Table 3, the interaction term was significantly negative in association with well-being (b = −0.43, *p* < 0.001), providing clear evidence supporting H3b.

**Table 3:** Moderating effects of need for privacy.

Variables	Model 1 (Social capital)	Model 2 (Well-being)
Constant	−0.09	−0.08
Social grooming	0.32***	0.37***
Need for privacy	0.05	−0.02
Social grooming*Need for privacy	−0.10	−0.43***
R^2^	0.13	0.21
Adjusted R^2^	0.13	0.2
*F*-value	13.44***	28.24***
Breusch–Pagan χ^2^	4.4	5
Breusch–Pagan *p*-value	0.036	0.17
VIF (Social grooming)	1	1.19
VIF (Need for privacy)	1.2	1.2
VIF (Interaction)	1.01	1.01

***, **, and * denote the significance at 1, 5, and 10% levels.

**Figure 3:**
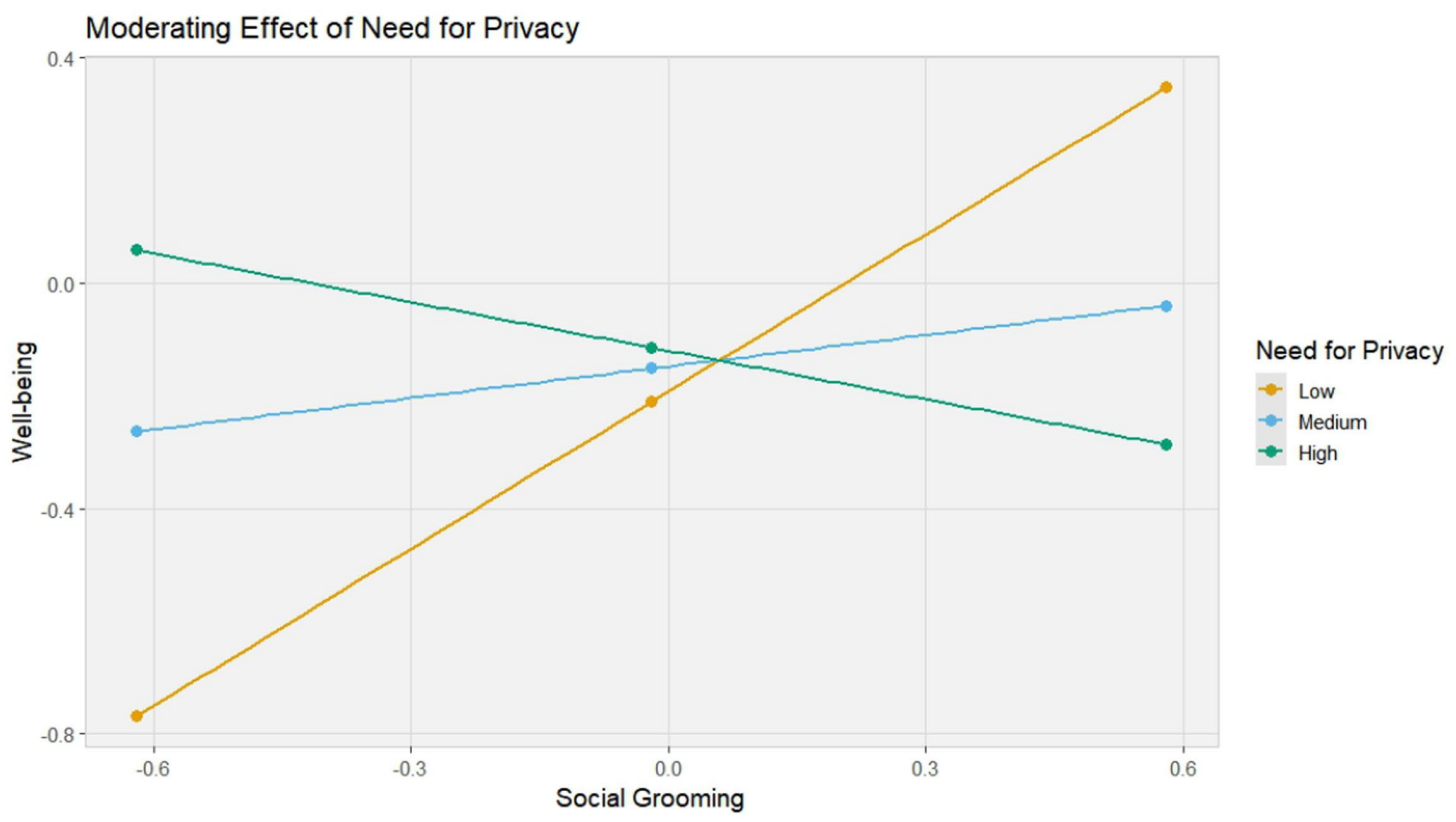
Simple slope.

Further analysis (Figure 3) revealed differential associations between social grooming and well-being depending on the level of need for privacy. Specifically, at lower levels of need for privacy (−1 SD), the relationship was robust and positive (b = 0.80, *p* < 0.001). At mean levels, the association remained significant but reduced in magnitude (b = 0.39, *p* < 0.001). However, at higher levels of need for privacy (+1 SD), the relationship diminished to statistical non-significance (b = 0.03, *p* = 0.71). Thus, the moderating effect of need for privacy is confirmed for the direct path, but not for the mediated pathway through social capital.

### 4.4 Model diagnostics and robustness

Multicollinearity diagnostics verified reliability, with all Variance Inflation Factor (VIF) values well within acceptable limits (<5; Tables 3, 4). Model fit indices (Table 5) indicated acceptable explanatory power, with R^2^ values of 0.13 (*F* = 45.42, *p* < 0.001) for social capital and 0.14 (*F* = 18.35, *p* < 0.001) for well-being.

**Table 4:** Multicollinearity diagnostics for mediation effect.

Dependent variable	Independent variables	Tolerance	VIF	Multicollinearity problem
Social capital	Social grooming	1	1	No
Well-being	Social grooming	0.87	1.15	No
	Social capital	0.87	1.15	No

Tolerance <0.1 or VIF >5 indicates serious multicollinearity issues.

**Table 5:** Fit and robustness analysis.

Outcome variable	R	R^2^	Adjusted R^2^	F-statistic	*p*-value	Breusch-Pagan Chi-Square	*p*-value	Robustness conclusion
Social capital	0.36	0.13	0.13	45.42	<0.001	4.4	0.036	Mild heteroscedasticity
Well-being	0.38	0.14	0.14	18.35	<0.001	4.29	0.12	No significant heteroscedasticity

Breusch–Pagan tests revealed slight heteroscedasticity for social capital (χ^2^ = 4.40, *p* = 0.036), while the well-being model showed homoscedasticity (χ^2^ = 4.29, *p* = 0.12). The application of heteroscedasticity-consistent (HC3) standard errors indicated robustness, as the significance and directionality of effects remained unchanged, underscoring the stability of our results.

### 4.5 Sensitivity analysis

**Figure 4:**
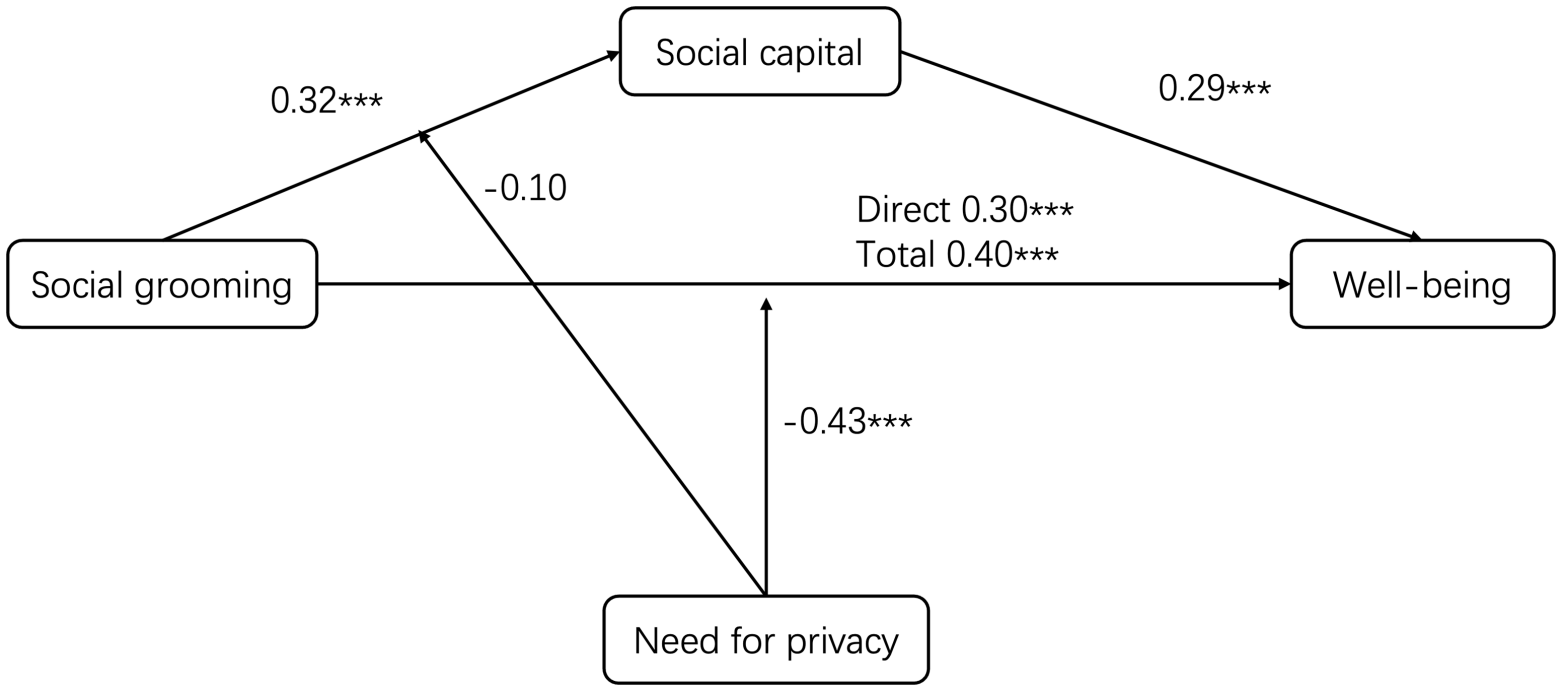
Effect path.

To further assess the robustness of the moderation findings (see Supplementary material), analyses were replicated by substituting the aggregate privacy measure with its distinct sub-dimensions (informational, psychological, and physical). Each replication yielded a consistently significant main effect of social grooming on well-being and reliably reproduced the moderating effect pattern across sub-dimensions (Supplementary material). Therefore, irrespective of operationalization as a unified or subdivided construct, need for privacy consistently moderated the direct relationship between grooming and well-being. The moderated mediation pathway is visually summarized in Figure 4.

## 5 Discussion

The results indicate that more frequent sharing of live photos as social grooming is positively associated with higher well-being. This association is partly explained by the role of social capital. However, the strength of this relationship depends on individuals’ need for privacy. Those with a stronger need for privacy experience reduced benefits from social grooming because their desire to connect socially conflicts with their wish to manage personal information. Consequently, these users receive fewer psychological benefits from their social interactions.

Even though users cannot visually edit live photos, they still manage impressions by selectively choosing their audience and deciding which moments to share. This selective disclosure clarifies why a higher need for privacy weakens—but does not fully eliminate—the association between social grooming and well-being. Although our study cannot establish definite causal conclusions, our findings suggest a plausible interpretation: users obtain greater social benefits from visual sharing when their privacy needs are satisfied within a closed social network.

Our findings differ from previous studies in two important respects. First, while prior studies mainly examined social grooming using traditional content forms such as text-based posts and static images (Lin et al., 2024; Liu and Yeo, 2024), we found that live photo–sharing on closed platforms produces comparable relational benefits despite reduced editing control. Second, in contrast to previous research that typically treated privacy as a situational factor or a cost–benefit calculation, our results indicate that the stable need for privacy systematically moderates the association between social grooming and well-being. By integrating Communication Privacy Management theory (Petronio and Child, 2020), which highlights dynamic boundary negotiation, with the Social Grooming Model (Lin, 2019), our study emphasizes privacy as an integral, ongoing component of relational interactions rather than a separate or purely contextual concern. Our findings thus align with broader evidence that self-disclosure and social support enhance well-being (Faelens et al., 2021; O’Day and Heimberg, 2021), but also demonstrate how individual differences in privacy preferences may moderate these benefits.

People continuously negotiate privacy boundaries to achieve a desired comfort level in social grooming. In this view, privacy regulation corresponds with inner needs for autonomy, intimacy, and safety, not just external calculations. For example, when users share a live photo with a closed friends group on social media, they are actively managing the boundary between public and private spheres: the audience is deliberately restricted to an inside circle, satisfying a psychological need for connection within a trusted space while keeping other audiences out. Such selective sharing illustrates privacy as an ongoing boundary negotiation aligned with personal comfort and relational norms. The ephemeral and group-delimited context of closed-network (Ma et al., 2021; Zhang et al., 2022) live photo–sharing often lowers users’ guard and encourages disclosure because the controlled boundaries (e.g., time-limited visibility and chosen recipients) assure individuals that their inner need for privacy remains balanced. This type of privacy on social media provides users with relational, perceptible, and contextually relevant affordances (Ronzhyn et al., 2023). Thus, communication privacy management theory reconceptualizes privacy in closed social media as a dynamic process of aligning sharing behaviors with one’s evolving psychological and interpersonal boundaries, moving beyond the static cost–benefit logic of privacy calculus toward a richer understanding of privacy as boundary fulfillment (Altman, 1975; Petronio, 2013; Petronio and Child, 2020).

The moderating role of the need for privacy in live photo–sharing may also be connected to shifting privacy boundaries. A live photo captures both visual and audio details at a single moment, yet its implications for privacy can change as the surrounding social context evolves. Thus, a live photo functions like a time capsule, potentially opened by future audiences under very different relational circumstances. Participants with a higher need for privacy reported fewer well-being benefits, indicating their sensitivity not only to who currently views their live photos but also to who might revisit, forward, or reinterpret them later. In other words, dynamic images serve as durable social artifacts whose privacy boundaries must adapt continuously to evolving relationships. When boundary adjustments lag behind relational changes, users experience increased psychological costs and reduced grooming benefits. Consequently, design features allowing users to retroactively adjust visibility, such as audience removal after posting or adjustable expiry timers, may be particularly beneficial for users with high privacy needs, enabling them to comfortably engage in social sharing without sacrificing relational rewards.

By demonstrating that the need for privacy weakens—but does not eliminate—the association between social grooming via live photo sharing and well-being on WeChat Moments, our study provides a nuanced understanding of privacy boundary management under spontaneous, non-editable sharing. This pattern is likely not limited to WeChat alone. As short-form live videos, multi-camera streams, and other real-time formats continue to expand across Instagram, TikTok (ByteDance, Beijing, China), YouTube (Google LLC, Mountain View, CA, USA), and emerging mixed-reality platforms, designers, regulators, and users globally will likely face similar tensions between authenticity and privacy control.

This theoretical integration makes three contributions. First, the study demonstrates that social grooming with dynamic visuals (i.e., live photos) is positively associated with well-being through social capital, thereby extending the Social Grooming Model beyond text-based and static visual content. Second, by embedding Communication Privacy Management within the Social Grooming Model and introducing the need for privacy as a dispositional moderator, the study moves privacy considerations from the periphery of cost–benefit calculations into the core of everyday relational exchanges. Third, the resulting moderated mediation model integrates relational rewards and privacy boundary motivations, explaining how they jointly shape the psychological outcomes of routine mobile sharing on closed social networks.

This study also has practical implications. For users, adjusting audience scope, post-capture review, and visibility duration according to personal privacy comfort can maximize relational benefits while minimizing boundary tensions. For platform designers, prioritizing features such as granular privacy controls, retroactive audience removal, adjustable expiry timers, and default closed-circle sharing modes will help users with higher privacy needs participate comfortably and without anxiety.

Several limitations should be considered when interpreting our findings. First, because this study relied on a single cross-sectional questionnaire for data collection, all variables were self-reported and collected at a single time point, preventing causal inference and raising the possibility of common-method bias. Second, we did not include measures of perceived authenticity or felt realness, even though live photo–sharing was theorized to foster more genuine self-presentation; omitting this construct leaves the psychological mechanism linking disclosure to well-being underspecified. Third, the survey offered no manipulation check for the core stimulus, live photos, so we could not confirm that participants actually viewed these images as more dynamic, less editable, or more relationally potent than static photographs. Fourth, the study sample was drawn exclusively from WeChat Moments, a closed friends list environment; platform-specific affordances (e.g., inability to repost publicly) may limit the applicability of our results to open or follower-based networks. Finally, all grooming behaviors were measured retrospectively and may be colored by recall or social-desirability bias.

Addressing these limitations calls for a multi-method approach. Experiments that systematically vary content format (static vs. live), curation level (edited vs. spontaneous), and privacy settings can test whether live photo dynamism and perceived authenticity jointly mediate social capital and well-being outcomes. Longitudinal or experience-sampling designs would help to clarify temporal ordering and reduce recall bias while incorporating physiological or behavioral indicators of authenticity could triangulate self-reports. Cross-platform replications on Instagram Close Friends, Snapchat private stories, and TikTok LIVE may reveal whether boundary-management dynamics differ in follower-based or algorithmically amplified contexts, and cross-cultural samples can probe how normative disclosure expectations shape the privacy–reward calculus. Finally, future studies might examine discrete emotional responses to live photo–sharing, such as pride, embarrassment, or nostalgia, to build a more granular account of how spontaneous visual disclosures translate into psychological and relational benefits.

## 6 Conclusion

Live photo-based social grooming on a closed platform is associated with higher user well-being, through greater social capital. Users who frequently share dynamic visual content and respond to others’ posts generally report experiencing greater. However, this relationship varies depending on individual privacy preferences. Users with stronger need for privacy experience smaller benefits, indicating that privacy concerns can limit the positive effects of social grooming. By combining the Social Grooming Model with Communication Privacy Management, and including need for privacy as a moderator, the study highlights two aspects of modern digital interactions: users regularly share personal content to strengthen relationships while carefully managing their boundaries. Platforms offering easy-to-use audience controls, post-sharing review features, and adjustable visibility options can help users comfortably turn brief moments into lasting social connections without sacrificing their sense of personal privacy.

## Data Availability

The raw data supporting the conclusions of this article will be made available by the authors, without undue reservation.
