# Supplementary material for: The effect of social grooming via live photo-sharing on well-being: the mediating role of social capital and moderating role of the need for privacy
Source: Front Psychol. 2025 Jul 11;16:1627455. doi: 10.3389/fpsyg.2025.1627455 (PMC12289657; doi:10.3389/fpsyg.2025.1627455)
Supplement: Supplementary file 1 [file Table_1.docx]

Supplementary Material

# Supplementary **Descriptive Statistics for Each Item**

## Social Grooming

**Table1: Social Grooming**

| Item | Question | Mean | SD |
| --- | --- | --- | --- |
| 1 | Share personal emotions through live photos (e.g., happiness or sadness) | 3.12 | 0.83 |
| 2 | Share daily events through live photos (e.g., activities, meals) | 3.24 | 0.77 |
| 3 | Express opinions on controversial issues through live photos | 2.44 | 0.99 |
| 4 | Discuss noncontroversial trending topics through live photos | 2.98 | 0.92 |
| 5 | Respond to others' live photos (liking, commenting, emojis) | 3.31 | 0.79 |

***Note:*** *composite Score (average): M = 3.02, SD = 0.64*

## **Social Capital**

**Table 2: Social Capital**

| Item | Question | Mean | SD |
| --- | --- | --- | --- |
| 1 | Several people I trust to help solve my problems | 3.81 | 0.89 |
| 2 | Comfortable discussing intimate problems | 3.94 | 0.8 |
| 3 | People available to talk to when lonely | 3.91 | 0.77 |
| 4 | Interaction makes me want to try new things | 4.03 | 0.77 |
| 5 | Interested in opinions of people unlike me | 3.86 | 0.84 |
| 6 | Talking with others makes me curious about other places | 3.86 | 0.69 |

***Note:*** *composite Score (average): M = 3.90, SD = 0.56*

## **Need for Privacy**

**Table 3: Informational Need for Privacy**

| Item | Question | Mean | SD |
| --- | --- | --- | --- |
| 1 | Dislike live photos publicly accessible | 3.22 | 0.94 |
| 2 | Prefer minimal personal information in live photo posts | 3.03 | 1.04 |
| 3 | Not everyone needs details in my live photos | 3.36 | 1.06 |
| 4 | Prefer live photos visible only to specific people | 3.08 | 1 |
| 5 | Aspects of life not shared in live photos | 2.98 | 1 |
| 6 | Uneasy with others revealing private details | 2.82 | 1.1 |
| 7 | Hard sharing personal ideas in live photos | 2.85 | 1.05 |
| 8 | Dislike constant private posts | 2.62 | 0.99 |
| 9 | Dislike strangers viewing my live photos without permission | 3.12 | 1.02 |
| 10 | Uncomfortable when others enter room/check photo album unannounced | 3.36 | 1.23 |
| 11 | Dislike being photographed in crowded locations | 3.2 | 1.15 |
| 12 | Dislike sudden appearances in live photos | 3.18 | 1.07 |

***Note:*** *composite Score (average): M = 3.07, SD = 0.66*

## **Well-being**

**Table 4: Well-being**

| Item | Question | Mean | SD |
| --- | --- | --- | --- |
| 1 | Feel closer to friends via live photo interactions | 3.36 | 0.91 |
| 2 | Generally satisfied with life | 3.47 | 0.96 |
| 3 | Generally satisfied with social life | 3.42 | 0.95 |
| 4 | Generally happy with current life | 3.5 | 0.95 |

***Note:*** *composite Score (average): M = 3.44, SD = 0.78*

# Supplementary **Sensitivity Analysis**

**Table 5. Moderating Effects of Need for Privacy**

| Variables | Model 1 (Informational) | Model 2 (Psychological) | Model 3 (Physical) |
| --- | --- | --- | --- |
| Constant | -0.09 (0.04) † | -0.08 (0.05) | -0.08 (0.05) |
| Social Grooming | 0.41*** (0.08) | 0.34*** (0.08) | 0.32*** (0.08) |
| Informational Need for Privacy | 0.05 (0.06) | – | – |
| Psychological Need for Privacy | – | -0.08 (0.05) | – |
| Physical Need for Privacy | – | – | -0.05 (0.04) |
| Social Grooming × Informational Need for Privacy | -0.42*** (0.07) | – | – |
| Social Grooming × Psychological Need for Privacy | – | -0.42*** (0.08) | – |
| Social Grooming × Physical Need for Privacy | – | – | -0.45*** (0.07) |
| R² | 0.19 | 0.2 | 0.23 |
| Adjusted R² | 0.18 | 0.2 | 0.22 |
| F-value | 20.98*** | 30.87*** | 22.88*** |
| Breusch-Pagan Chi-Square | 3.06 | 5.2 | 5.79 |
| Breusch-Pagan p-value | 0.382 | 0.158 | 0.122 |

*Notes:* **********, **, and * denote the significance at 1%, 5%, and 10% levels.*

**Table 6. Multicollinearity Diagnostics for Moderation Effect**

| Variables | Model 1 | Model 2 | Model 3 |
| --- | --- | --- | --- |
| Social Grooming | 1.19 | 1.12 | 1.10 |
| Informational Need for Privacy | 1.20 | – | – |
| Psychological Need for Privacy | – | 1.12 | – |
| Physical Need for Privacy (w3) | – | – | 1.09 |
| Social Grooming*Informational Need for Privacy | 1.01 | – | – |
| Social Grooming*Psychological Need for Privacy | – | 1.01 | – |
| Social Grooming*Physical Need for Privacy | – | – | 1.02 |

***Notes:*** *tolerance <0.1 or VIF >5 indicates serious multicollinearity issues*
